# Sulphate of Magnesia a Potent Remedy

**Published:** 1900-11

**Authors:** N. F. Howard

**Affiliations:** Dahlonega, Ga.


### SULPHATE OF MAGNESIA A POTENT REMEDY.

By N. F. HOWARD, M.D.

DAHLONEGA, GA.

Fifty years ago, when I began to practice medicine, if a case of puerperal fever, attended with peritonitis, had been treated by the profession with sulphate of magnesia and his female patient had died, the attending physician might have been subject to indictment for malpractice. At this time, if a patient were afflicted with the same disease, and treated by a physician *without* the use of Epsom salts, and said patient were to die, the physician would be liable to be indicted for malpractice, because he had *not* used sulphate of magnesia.

What a great change has taken place in the last fifty years in the treatment of this most dangerous fever, and what a simple medicine has become the most potent remedy used for the relief of said fever!

I do not remember who made the discovery of this important agent for the treatment of this fever.

I desire to ask this question, to wit: If Epsom salts is such a sure and potent remedy in the treatment of such a severe form of inflammation as puerperal peritonitis, why then can it not be trusted in the treatment of all other forms of inflammation the human body is heir to? In answer we state that we believe it can be so trusted, except where said inflammation is attended with some malignant disease. We know that a saturated solution of sulphate of magnesia, in either cold or warm water, applied to burns, will relieve the pain in a moment, if the burn is made by fire; if the burn is made by scalding water, the time required is a little longer, and if by steam the time is a little longer still before the pain is relieved. In one case of this kind it was nearly an hour. In all these forms of burns, and we have used it in all, we have found that the pain, fever, swelling, and inflammation have subsided and entire relief been given in a few hours, and that the wounds made by the burning can be afterwards treated with very simple remedies and soon restored to health—such remedies, for example, as vaseline, lime-water, mixed with linseed oil, castor oil, etc. Some I have treated solely with salts-water, and entire relief was given.

As I wrote in a communication March, 1894, which was published in the *Atlanta Medical Journal*, and stated other cases connected with the use of Epsom salts, and that my wife made the discovery by chance, in 1868 or 1869, I will further state that my test of the medicine since has confirmed all that I said in that communication.

I wish to add that soon after I wrote I concluded that if it would relieve inflammation and congestion of the soft tissue produced by burns, why then would it not relieve any and every form of inflammation of the human tissues that the body is subject to? I then began to apply the remedy in all such cases in my own person and that of others which came under my care; and it was prudent to do so. I was not disappointed in the results, as the remedy was fully as efficient in these injuries as it was for burns.

I will just add here that the salts applied to the parts suffering from the sting of a honey-bee, yellow-jacket, wasp, or hornet will relieve the pain at once. This I know by the actual use of the remedy.

I also wish to add that the use of this remedy has given satisfactory results when applied to injuries to my own person, such as bruises, contusions, lacerations, etc., that have occurred so many times in the last year or two on account of imperfect vision from glaucoma. These injuries have occurred nearly every day upon my lower extremities, hands, or face. In treating all these injuries I have used a solution of sulphate of magnesia with entire relief and restoration to health.

I wish to give two severe cases out of a great many in which I used this medicine with the happiest results.

Two years ago I was called to see Mrs. R., of our town, a healthy lady in middle life, who was suffering from an injury of her ankle-joint. Early in the morning she stepped off the piazza floor in haste, a distance of a foot or more, upon some object that turned under her foot and threw her down, so injuring the joint that the bottom of her foot turned inwards, making her think when she called for help and her husband came, that the ankle-joint was displaced. The foot was, by help, pulled back and kept in place. Late in the afternoon I saw her and found her suffering great pain. The foot, ankle, and leg up to the knee-joint were much swollen, and the swollen parts were very hot. She informed me that for most of the day she had been bathing the injured parts in a tub of warm water made strong with common salt, but had received no benefit thereby, but grew worse all the time, and was suffering more pain then than she had all the day.

I directed her to add a pound of Epsom salts to the water and continue the bathing until she received comfort and relief from the pain. This she did for two or three hours into the night, when she received relief. At 10 o'clock at night she wrapped up the parts in a cloth dipped in salts-water, and lay down to sleep and rested till morning, when she found that the swelling, pain and trouble had entirely disappeared, with only a slight soreness in the ankle-joint. She arose and dressed herself as usual, and walked about the house as if well.

Next morning about 8 o'clock, when I paid her a visit, I was not only surprised, but might say astonished, when I found my patient up and about, as I have stated, looking after the affairs of her house. It was one of the most severe sprains and bruises of the ankle-joint I had ever met with, and I did not expect recovery from this injury for several weeks. My patient remained up all day, with a little pain and soreness of the ankle-joint and foot, which parts she bathed again at night in Epsom-salts water for an hour or so, wrapping the parts in cloths dipped in salts-water and retiring at bedtime. With a good night's rest my patient had no further trouble from this injury.

The other case is this, to wit: About one year ago Mr. A, a mechanic, was working at the Consolidated mill, just outside the corporate limits of Dahlonega, his duty being to prepare for the erection of heavy stamps. While he was engaged in this work and was boring with a large auger, a heavy guide-post fell against him, broke the auger and carried Mr. A. some three feet downward to the floor, on which he fell upon one knee. The fall was a heavy shock to him and gave him much pain. He managed, however, to walk a hundred yards or so to a smith-shop and have his auger welded, and back to the mill with it and resume work for a short time. He then walked to town, the distance of a mile. Soon after his arrival I saw the patient. He is a man in the prime of life, weighs some hundred and ninety pounds and is in a state of good health. I found him suffering much with his knee, which had begun to swell. I found no fracture of bones of any part. The suffering seemed to be from pain in the knee-joint.

I advised him for the present to apply warm Epsom-salts water, and said that I would see him again presently; I did so at 9 o'clock P. M., and found him suffering greatly with pain in the knee-joint and the soft parts about the knee-joint were swollen and somewhat red. He had used the Epsom salts but little up to this time. We had a saturated solution of Epsom salts prepared of several gallons and the leg immersed in it and kept wet with it above the knee-joint for three hours.

At midnight the pain gave way, the swelling and heat had subsided and the patient had a good rest till morning, the knee being kept bathed continually through the night, and the severity of the trouble did not return, yet there was some soreness in the knee-joint for some weeks, which had to be favored by Mr. A. while at work, as he returned to his work next day, and continued to do so. His knee improved from day to day until it was entirely restored to health.

I was as much surprised at the result in this case as I was in that of Mrs. R., as I considered the case a very serious one and did not expect him to be up for some weeks.

### CASE OF ERYSIPELAS AND SCALP ERUPTION.

Last February an old gentleman, seventy-three years old, Mr. B., suffered with erysipelas of one foot and leg, which became much swollen. We applied a solution of sulphate of magnesia and glycerine to all the parts affected and repeated it every three hours. Also dipped cloths in the solution of salts and wrapped up with them the parts affected. In forty-eight hours the inflammation had subsided and the patient was convalescent and up and about.

This is the only case of this kind I have treated with the salts, and cannot say if repeated in another case the result would prove so successful.

I have treated several scalp eruptions with the salts, as eczema, etc., with entire success, by rubbing the medicine upon the scalp once a day.

